# Supplementary material for: Computed Tomography Angiography-Guided Study of the Superficial Femoral Artery Course in the Thigh and the Identification of Dangerous Zones for Lateral Femoral Surgical Approaches
Source: Medicina (Kaunas). 2025 Feb 28;61(3):441. doi: 10.3390/medicina61030441 (PMC11944110; doi:10.3390/medicina61030441)
Supplement: Supplementary file 1 [file medicina-61-00441-s001.zip › medicina-3484005-supplementary.pdf]

Pre-test

Table S1. t tests – Means: Difference between two independent means (two groups)

**Analysis:**	A priori: Compute required sample size		
**Input:**	Tail(s)	=	Two
	Effect size d	=	0.59
	α err prob	=	0.05
	Power (1–β err prob)	=	0.95
	Allocation ratio N2/N1	=	1
**Output:**	Noncentrality parameter δ	=	3.6370043
	Critical t	=	1.9759053
	Df	=	150
	Sample size group 1	=	76
	Sample size group 2	=	76
	Total sample size	=	152
	Actual power	=	0.9509014

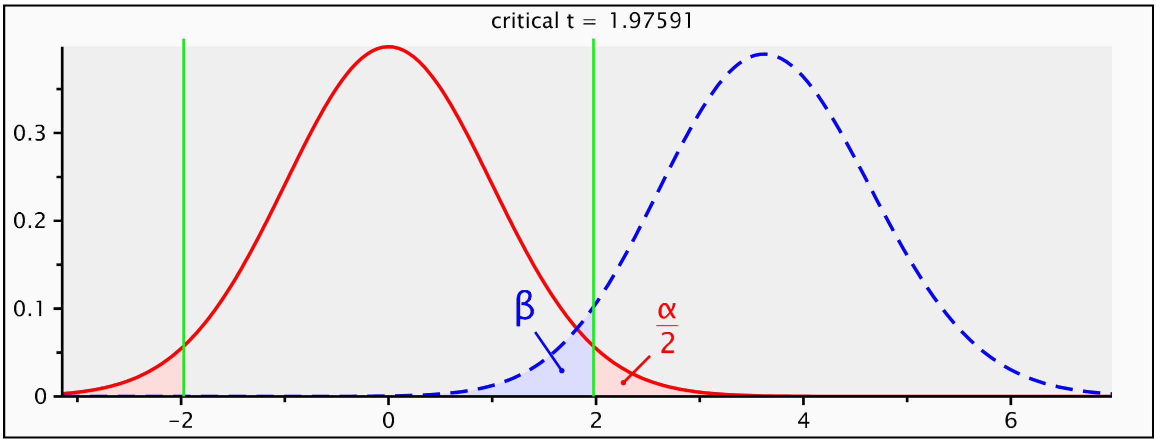

Figure S1. Adequate sample size and power analysis graphs created using G*Power version 3.1.9.7.

Test result for our study

Table S2. t tests – Means: Difference between two independent means (two groups)

**Analysis:**	A priori: Compute required sample size		
**Input:**	Tail(s)	=	Two
	Effect size d	=	0.59
	α err prob	=	0.05
	Power (1–β err prob)	=	0.95
	Allocation ratio N2/N1	=	1.3
**Output:**	Noncentrality parameter δ	=	3.6298500
	Critical t	=	1.9756939
	Df	=	152
	Sample size group 1	=	67
	Sample size group 2	=	87
	Total sample size	=	154
	Actual power	=	0.9502063

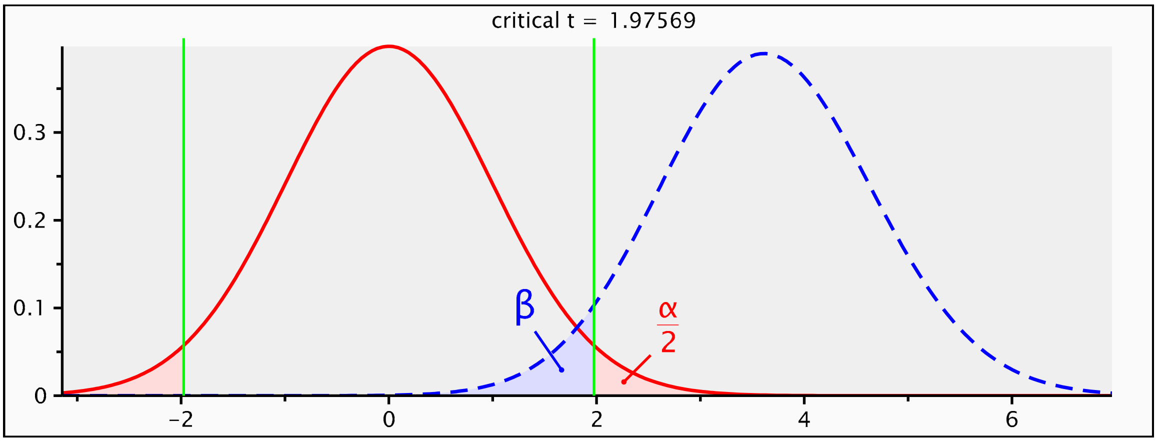

Figure S2. Adequate sample size and power analysis graphs created using G*Power version 3.1.9.7.

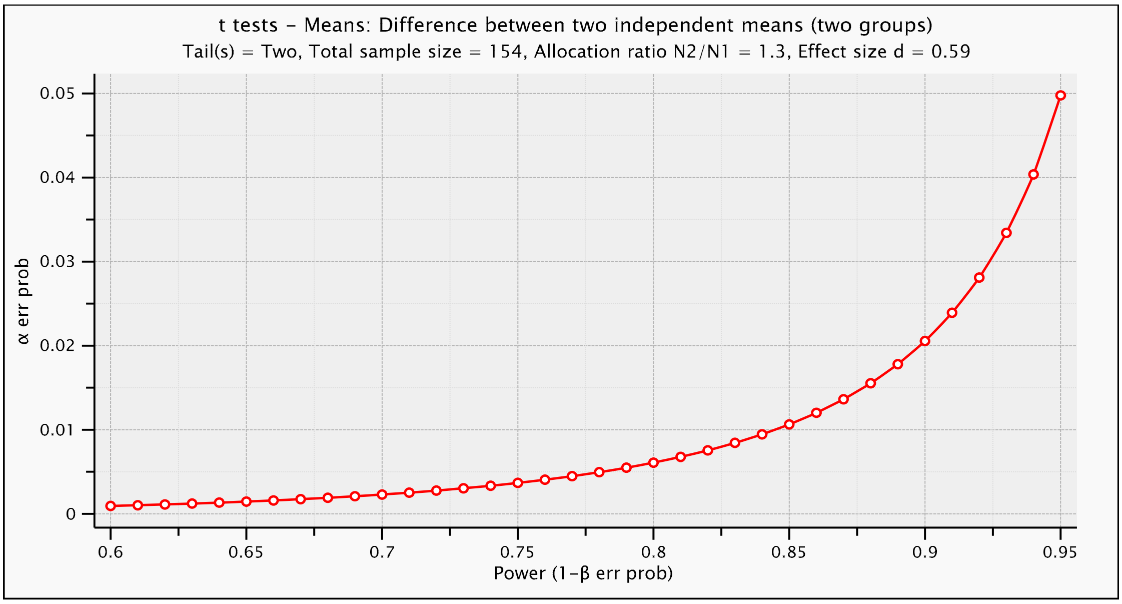

Figure S3. Adequate sample size and power analysis graphs created using G*Power version 3.1.9.7.

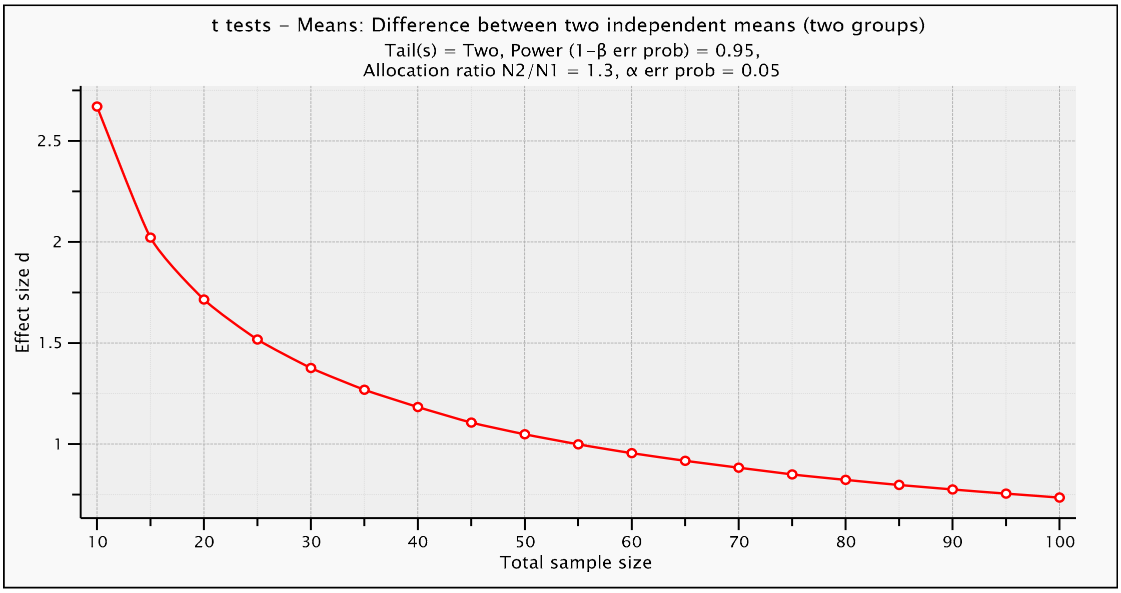

Figure S4. Adequate sample size and power analysis graphs created using G*Power version 3.1.9.7.

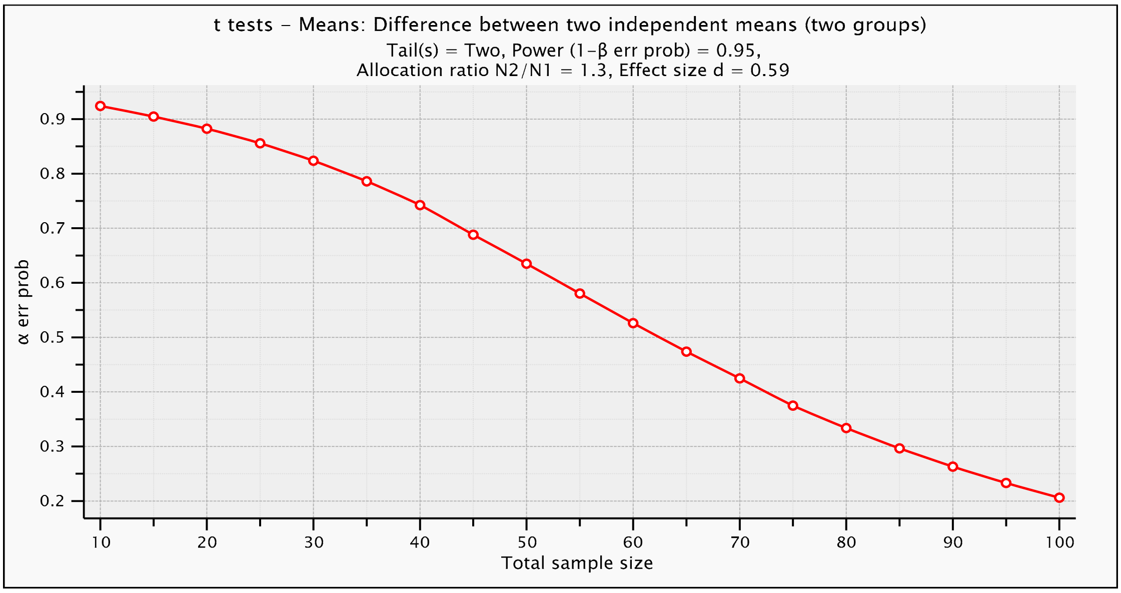

Figure S5. Adequate sample size and power analysis graphs created using G*Power version 3.1.9.7.

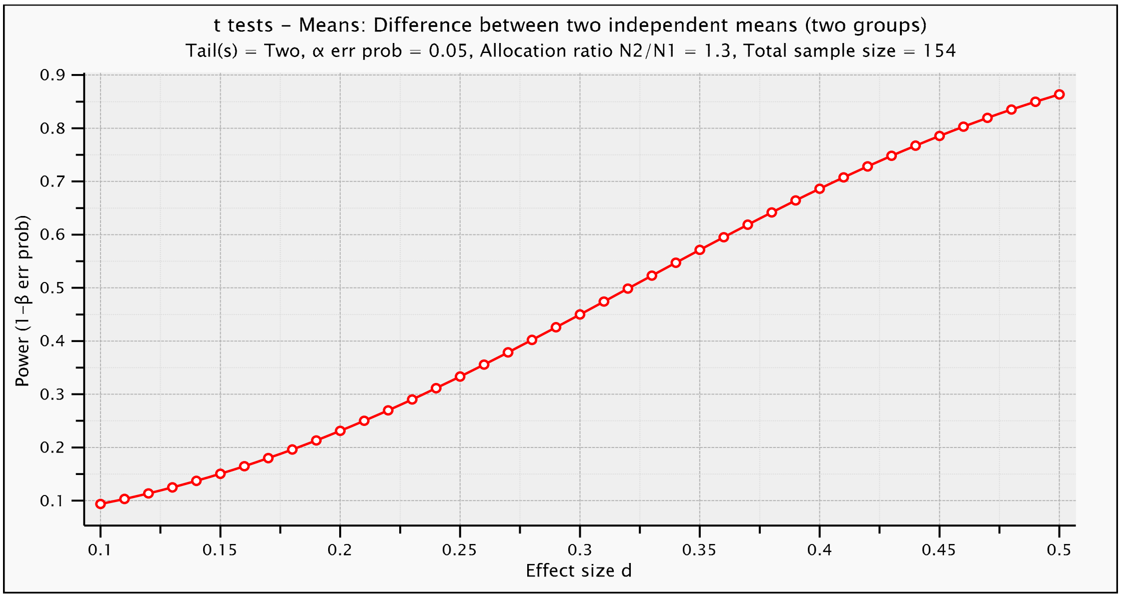

Figure S6. Adequate sample size and power analysis graphs created using G*Power version 3.1.9.7.
